# Pattern recognition based on machine learning identifies oil adulteration and edible oil mixtures

**DOI:** 10.1038/s41467-020-19137-6

**Published:** 2020-10-23

**Authors:** Kevin Lim, Kun Pan, Zhe Yu, Rong Hui Xiao

**Affiliations:** 1 Wilmar International Limited, WIL@NUS Corporate Lab, Center for Translational Medicine, 14 Medical Drive, Singapore, Singapore; 2 Yihai Kerry Arawana Oils, Grains & Food Co., Ltd, Arawana Building, No. 1379 Bocheng Road, Pudong New District, Shanghai, China

**Keywords:** Fatty acids, Machine learning, Statistical methods, Agriculture, Technology

## Abstract

Previous studies have shown that each edible oil type has its own characteristic fatty acid profile; however, no method has yet been described allowing the identification of oil types simply based on this characteristic. Moreover, the fatty acid profile of a specific oil type can be mimicked by a mixture of 2 or more oil types. This has led to fraudulent oil adulteration and intentional mislabeling of edible oils threatening food safety and endangering public health. Here, we present a machine learning method to uncover fatty acid patterns discriminative for ten different plant oil types and their intra-variability. We also describe a supervised end-to-end learning method that can be generalized to oil composition of any given mixtures. Trained on a large number of simulated oil mixtures, independent test dataset validation demonstrates that the model has a 50^th^ percentile absolute error between 1.4–1.8% and a 90^th^ percentile error of 4–5.4% for any 3-way mixtures of the ten oil types. The deep learning model can also be further refined with on-line training. Because oil-producing plants have diverse geographical origins and hence slightly varying fatty acid profiles, an online-training method provides also a way to capture useful knowledge presently unavailable. Our method allows the ability to control product quality, determining the fair price of purchased oils and in-turn allowing health-conscious consumers the future of accurate labeling.

## Abstract (web summary)

Fraudulent adulteration of edible oils is based on the fact that their characteristic fatty acid profile can be mimicked with mixtures of other oil types. Here, the authors use a deep learning method to uncover fatty acid patterns discriminative for ten different plant oil types and to discern composition of mixtures.

## Introduction

Over the last century there has been a significant shift to using plant oils rather than animal fats in human diets. This shift has prompted numerous scientific investigations on the characterization of fatty acid profiles in the commonly consumed plant oils culminating in a rather large database^1^. However, notwithstanding our knowledge on the fatty acid profiles of specific plant oil types^2,3^, it has not been possible to deduce and identify a plant oil when given the fatty acid profile. In addition, once two or more plant oils are mixed, the resulting fatty acid profile also changes. In many cases, the fatty acid profile of a blended oil mixture can mimic that of a high-quality product. Taking advantage of this possibility, counterfeiters sell low-value blended oils as high-value products and profit from the price difference; moreover, they even change the blending formulae in response to changes in market prices owing to macroeconomic factors.

The UC Davis olive center reported that up to 69% of California olive samples labeled as extra virgin olive oil failed to meet USDA standards mainly, because of adulteration with cheaper refined olive oil amongst many other reasons^4^. In another independent report, 82% of avocado oil sold in the US market were either expired or adulterated^5^. In Taiwan, olive oil sold to consumers was reported to be 98% palm oil^6^. Such adulterations led to the degradation of sensory quality without significant health effects. Moreover, in some cases, the consequence of adulteration can also pose public health issues. For example, in India, mustard oil adulterated with argemone oil has been reported to cause epidemic dropsy^7^. Adulterations of edible oils are more rampant in countries where regulatory bodies lack the resources and methods to assess and verify the quality of marketed products raising issues of food safety and security. Recent trend to promote nutritional guidance at a personal level further highlights the need for accurate and unadulterated labeling^8^. For example, a consumer who is at a higher risk of cardiovascular disease may prefer to have specific dietary fatty acid requirements provided by specific plant oil types. The ability to detect oil adulteration therefore has both economic and health consequences.

A number of chemometric methods to detect adulterated oil using fatty acid profiles has been described^9–12^. Usually these methods deal with a simple mixture of 2–3 oil types and are often qualitative in nature (e.g., PCA). When the methods provide quantitative information relating to the volume proportion of the major oil types (e.g., PLS1), the results would further require human interpretation when more than two oil types are being considered. This is because prevalent chemometric methods for quantifying oil adulteration assume a single target variable by considering the volume proportions of a mixture of only two oils. Even so, extending this simple methodology to a combination of mixtures of more than two oils, requires additional intermediate preprocessing because such models ignore correlations between multiple targets. Whereas single-target variable prediction chemometric methods also exist, extending chemometric methods to encompass multiple-targets (PLS2) resulted in a decrease in accuracy, when these two-oil mixtures are considered in a generalized context of multiple possible oil combinations (Supplementary Tables 1–4). Consequently, these models are seldom implemented in a practical setting due to the lack of an end-to-end solution.

Here, we report a machine learning method that is able to uncover discriminative fatty acid profile patterns between oil types. We use an unsupervised model to further identify subclusters within the larger oil types and narrow down specific fatty acid differences between the subclusters. Using the insights discovered, in silico simulation of oil mixtures provides a large training examples for a supervised end-to-end deep learning model to decipher quantitative compositional status of the oils. Independent blind test set based on genuine oil mixtures demonstrates the model′s ability to learn and generalize to real-world mixtures. To improve the general applicability of the model, we describe an online machine learning method^13^ that updates the model based on fatty acid profiles of future oils. This ability to continuously extend the utility of the model to oils with new fatty acid profiles provides a valuable unified resource for the industry to establish common standards for food safety and security.

## Results

### The fatty acid profiles of ten edible oils types

**Fig. 1:**
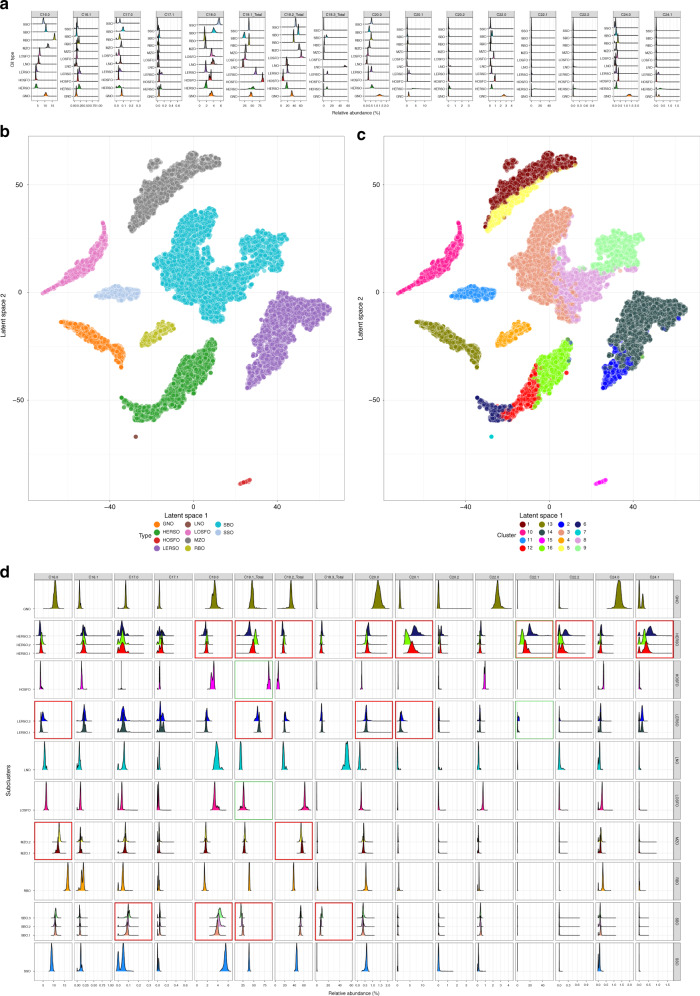
Characterization of ten edible oil types. **a** The fatty acid distributions of ten plant oils, groundnut (GNO), high-erucic acid rapeseed (HERSO), high oleic acid sunflower (HOSFO), low-erucic acid rapeseed oil (LERSO), linseed (LNO), low-oleic acid sunflower (LOSFO), maize (MZO), ricebran (RBO), soybean (SBO) and sesame seed (SSO), in relative abundance stratified across 16 fatty acids C16:0 to C24:1. **b** Ten plant oils visualized in the latent space. Clusters are colored by the oil types. **c** GMM clusters colored in the same latent space. **d** The empirical distributions of the subclusters labeled with numerical suffices, the major fatty acid changes are highlighted in red boxes.

We extracted lipids from 19,583 oil samples covering ten edible oil types obtained from Wilmar production plants: groundnut oil (GNO), high-erucic acid rapeseed oil (HERSO), high-oleic acid sunflower oil (HOSFO), low-erucic acid rapeseed oil (LERSO), linseed oil (LNO), low-oleic acid sunflower oil (LOSFO), maize oil (MZO), rice bran oil (RBO), soybean oil (SBO), and sesame oil (SSO) (Table 1). Fatty acids were derivatized and identified as fatty acid methyl esters (FAMES) by gas chromatography with flame ionization detector (GC-FID), which allowed the identification and quantification of at least 18 different fatty acids. The availability of this large data set enabled us to study the variation between and within the oil types. Comparison of fatty acid profiles among different oil types showed the presence of predominant fatty acids in some oil types (Fig. 1). For example, groundnut oil exhibited unique C20:0 and C22:0 profiles, high-oleic acid sunflower oil exhibited strong C18:1 profiles and linseed oils exhibited unique C18:3 profiles (Fig. 1a). However, we also noted considerable overlap in fatty acid profiles as well as fatty acid abundances across many other oil types. In addition, the presence of multiple modes across the fatty acid profiles suggested pattern heterogeneity and possible resolution of each oil species into subtypes (Fig. 1a). Subsequent data visualization using state-of-the-art dimensionality reduction algorithm^14^ supported this hypothesis as some large oil groups were found to disperse into smaller islands (Fig. 1b). By contrast, traditional chemometric methods are not able to clearly resolve all the oil clusters (Supplementary Fig. 1).

**Table 1:** Sample information.

Sample statistics (Number of samples)	
Groundnut (GNO)	1171
High-erucic acid rapeseed oil (HERSO)	2727
High-oleic acid sunflower oil (HOSFO)	169
Low-erucic acid rapeseed oil (LERSO)	3454
Linseed oil (LNO)	55
Low-oleic acid sunflower oil (LOSFO)	1319
Maize oil (MZO)	2658
Ricebran oil (RBO)	609
Soybean oil (SBO)	6728
Sesame oil (SSO)	693
Total	19,583

We extensively validated this hypothesis by fitting a Gaussian mixture model (GMM) on the data, assuming that the data points can be generated from a mixture of finite number of Gaussian distributions. Results from the expectation–maximization algorithm were further optimized by Bayesian Information Criterion, producing a total of 16 clusters (Fig. 1c). The clustering was mapped back to the dimensionality reduced latent space and the results largely corroborated the identities of the ten pure oil types. The identities inferred by the unsupervised GMM model achieved a precision and sensitivity rates of 99.9%, assuming that some of the oil types were divided into smaller subclusters (Table 2). As a consequence, we obtained 16 subclusters from ten larger clusters with samples in the same subcluster spatially colocalized in the dimensionally reduced embedding. This colocalization suggested that the Euclidean distance in the original fatty acid space within subclusters was smaller than across the subclusters, further supporting the idea that the heterogeneity previously observed was a result of specific fatty acid differences.

**Table 2:** Precision and sensitivity of GMM clusters.

	GNO	HERSO	HOSFO	LERSO	LNO	LOSFO	MZO	RBO	SBO	SSO	Sensitivity
1	0	0	0	0	0	0	1798	0	0	0	1
2	0	5	0	615	0	0	0	0	0	0	0.992
3	0	0	0	0	0	0	0	0	3263	0	1
4	0	0	0	0	0	0	0	609	0	0	1
5	0	0	0	0	0	0	860	0	0	0	1
6	0	570	0	0	0	0	0	0	0	0	1
7	0	0	0	0	55	0	0	0	0	0	1
8	0	0	0	0	0	0	0	0	2001	0	1
9	0	0	0	0	0	0	0	0	1464	0	1
10	0	0	0	0	0	1319	0	0	0	0	1
11	0	0	0	0	0	0	0	0	0	693	1
12	0	752	0	0	0	0	0	0	0	0	1
13	1171	0	0	0	0	0	0	0	0	0	1
14	0	1	0	2831	0	0	0	0	0	0	0.999
15	0	0	169	0	0	0	0	0	0	0	1
16	0	1399	0	8	0	0	0	0	0	0	0.994
Precision	1	0.998	1	0.998	1	1	1	1	1	1	

To further uncover patterns described by the within-oil subclusters, we compared the GMM parameters with the empirical distributions of the subclusters (Fig. 1d). We used the high-oleic variant of sunflower oil as a positive control as it was markedly higher in the C18:1 content compared to the low-oleic variant. Similarly, the high-erucic acid variant of rapeseed oil was more pronounced in C22:1 compared to the low-erucic acid variant (high-lighted green boxes). These observations supported the idea of subclusters, as the high-oleic and high-erucic acid oils can considered as originating from a single cluster, diverging as a consequence of evolution or selective breeding^15–17^. In fact, the high-erucic acid rapeseed and low-erucic acid rapeseed oils could be further separated into three and two subclusters, respectively. The subcluster HERSO.2, which was spatially closer to the LERSO clusters, contained fatty acids (C18:1, C20:1) with abundances resembling those of the LERSO types (Fig. 1d), suggesting that erucic acid was not the only discriminative feature of the two rapeseed oil varieties. Within the other subclusters, SBO.1, SBO.2, and SBO.3 displayed a coordinated shift in fatty acids C18:0 and C18:1. This result is consistent with published reports of a FAD2 mutant^18^ in which reducing C18:1 levels were correlated with increasing C18:0 levels. Some shifts in abundance across the subclusters were also visible with other uncommonly profiled fatty acids, e.g., C17:0 (Fig. 1d, highlighted red boxes). Taken together, our results showed that these oils are phenotypically different with changes in multiple fatty acids. Whether the differences can be attributed to geographical origins or due to the use of different processing methods remain to be investigated.

### Simulation of oil mixtures

The problem of oil mixtures has combinatorial roots. For any **n** types of oil, there are **2**^**n**^ possible combinations to describe the compositional makeup of the oil mixtures. To enumerate the quantitative value of these compositions we only considered discrete mixing at 1% steps. Then, this problem can be reduced into another combinatorial problem of fitting 100 indistinguishable balls into **n** identifiable baskets, commonly referred to as weak composition in mathematics. To assign each of the 100% into *n* oils, requires $$\left( {\begin{array}{*{20}{c}} {100\,+\,{\boldsymbol{n}} - 1} \\ {100} \end{array}} \right)$$ combinations. For the space of ten different oil types, this works out to be about 4.26 trillion, making it an intractable problem. Notwithstanding this, each oil type also has its own biological variability thereby enlarging the total space even more.

Fortunately, because of the insights and patterns discovered, the parameters learned can be used to simulate oil samples from a multinomial distribution. To approximate the compositional space, we first sampled pure oils from the GMM parameters and the quantitative compositional ratios through a Dirichlet distribution and combined the fatty acid profiles by a linear combination. By this method, an infinite number of training samples could be generated for learning the composition of an unknown oil mixture. This in silico method of sample generation prompted the question whether the biological variance of the pure oil types propagated through the oil mixtures presented itself as a noise or a consistent and generalizable signal. The latter would be important for constructing a robust machine learning model that is practical for real-world scenarios.

**Fig. 2:**
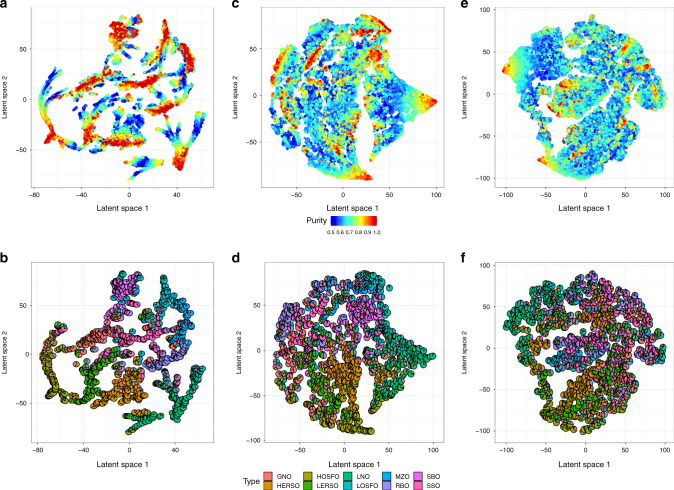
The latent spaces of two-way, three-way, and four-way oil mixtures. GMM simulated pure oil samples were mixed by linear combination of ratios drawn from a Dirichlet distribution and visualized in the latent space. Gradient legend (blue to red) used for (**a**–**c**), denoting the purity of oil measured against the major oil. Ten colors were used (**d**–**f**) to represent relative proportion of mixtures in the pie charts. **a**, **b** Ten epicenters in the two-way mixtures describe the ten edible oils in the purest form (red). The purity shifts to 50% (blue) as two oils are mixed in equal proportions, and then shifting back to red, in the epicenter of another pure oil type as indicated by the pie charts. **c**, **d** Three-way and **e**, **f** four-way mixtures exhibit the same patterns, even though the epicenters become fuzzier due to the complexity of mixtures.

To investigate data patterns from the simulated oil mixtures, we compared about 100,000 simulated oil mixtures across the ten oil types. In the latent space, red epicenters denoted oils that were pure and blue regions denoted severely adulterated oil mixtures. The pie charts denoted the compositional arrangements of the oil mixtures (two-mixtures Fig. 2a, b; three-mixtures Fig. 2c, d; four-mixtures Fig. 2e, f). Most oil type started off at their respective epicenters and gradually merged with other epicenters depicting the fact that the purity of oil transitioned from one type to another. We observed that as the complexity of the mixtures increased, i.e., from two-mixtures to four-mixtures the same pattern was preserved albeit some epicenters became fuzzy. This simulation result suggested that broad mixture trends can be captured by further supervised modeling, and larger errors were expected for complex mixtures.

### Deep learning model of oil composition

**Fig. 3:**
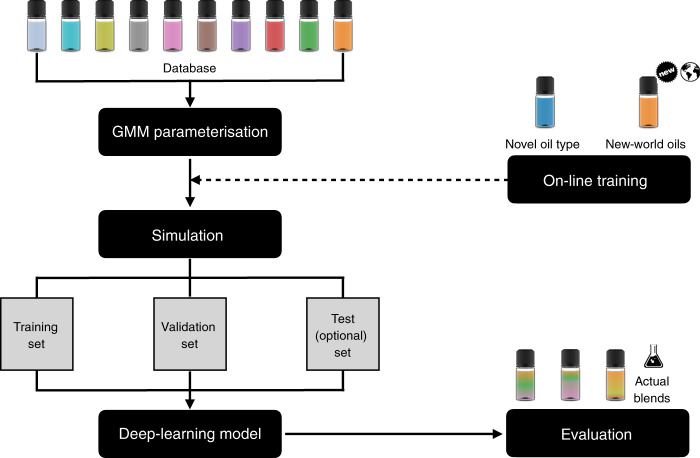
Machine learning schematic. The deep learning model was constructed with inputs simulated from priors learned with GMM. Simulation also allows multiple independent validation and test sets to be constructed for model optimization. Model evaluation is performed with real-world oil mixtures. Model deviation from the ground truth can be improved with an online-training process that generates new data based on novel or new-world oils to update model parameters.

**Fig. 4:**
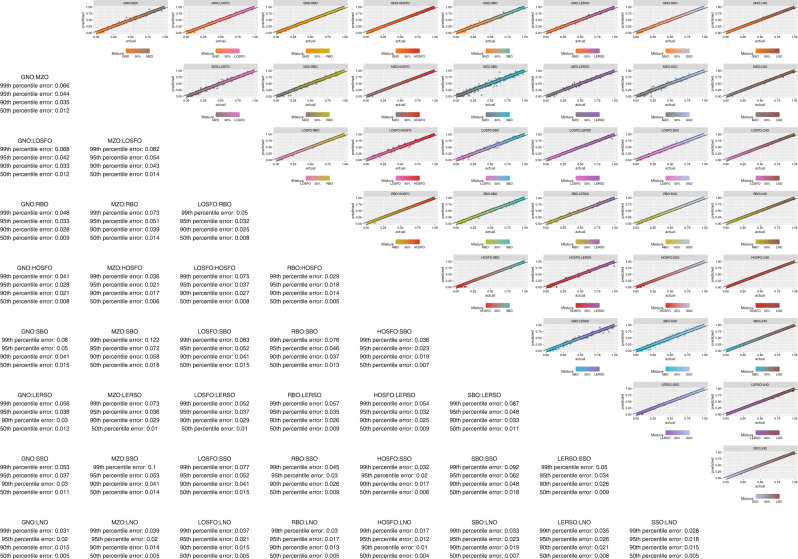
Deep learning results for two-way oil composition. All combinations of two-way mixtures, showing actual ratios against predicted ratios. The black lines indicate 5% margin of absolute error. Percentile absolute error is indicated in the lower triangle.

Recently, deep neural networks have been applied successfully to solve biological problems^19^. Compared to traditional chemometric methods they are more accurate and generalizable to unseen data^20^. Therefore, we generated a total of 12 million oil mixtures for construction and evaluation of the deep learning neural network model. A schematic describing the machine learning workflow is shown in Fig. 3. For a fatty acid profile of any unknown oil, the model must be able to determine not only the compositional arrangement across the oil types, but also have only small enough errors from the ground truth mixing ratios. To understand the performance better, we stratified the results according to the composition of 36 different possible two-mixtures (note that even though the data is visualized this way, the model is agnostic to this and must independently elucidate the oil composition without confusing other possible types). For each stratification, we summarized the results in terms of absolute error statistics based on the test set, e.g., the 50th percentile refers to the median absolute error (half of the test population has lower/higher than this absolute error). Our results showed that in most mixture types, the median absolute error is between 0.4 and 1.5%. The 90th percentile absolute error is between 1-5.8%, and can be a better gauge of generalizability (i.e., 90% of the test samples will have less than this absolute error), when a much stringent requirement is imposed on the predicted values for real-life quality control purpose (Fig. 4). In some cases where oil mixtures were harder to differentiate, higher margins of error were found, e.g., soybean and sesame oil mixtures had a 99th percentile absolute error of 9%. By contrast, traditional chemometric models trained by partial-least squares (PLS2) to differentiate the ten different oil types had errors ranging between 2.6 and 21.5% at the median and 9.1 and 40.1% at the 90th percentile. (Supplementary Fig. 2). This finding further demonstrates that the deep learning model has more power to generalize and discriminate unknown oil types based on fatty acid profiles.

**Fig. 5:**
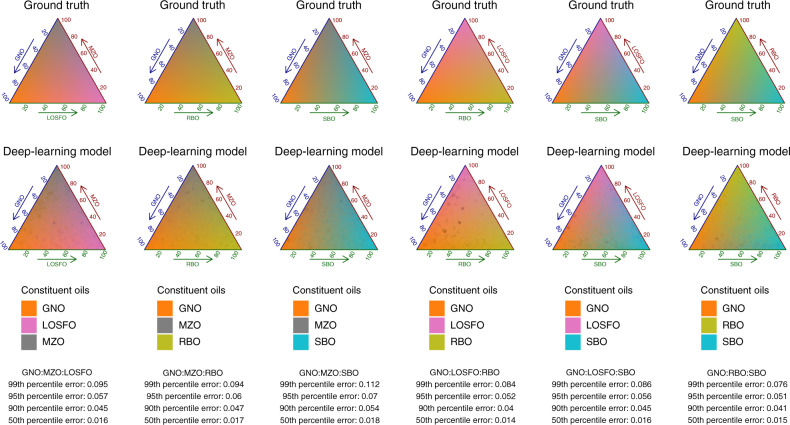
Deep learning results for three-way oil composition common to groundnut adulterants. The ground truth represented by the Gibb′s triangle, each point in the triangle correspond to exact ratios between three oils that sum to unity (top). The colors are the triangle represent the purity and the shift in color represents a mixture depending on the ratios between the other two oils. Results predicted by the three-way deep learning model are colored based on the relative ratios, colors that are closer to the ground truth have lower errors (middle). Percentile absolute errors (bottom).

A single model that can quantify any two-way mixtures without any prior knowledge to its composition can address the practical problem of simple oil adulteration. Generalizing the two-way model to three-way and other complex mixtures would also allow quick identification of these adulterants. We further demonstrated the general applicability of the deep learning model in two ways. First, independently generated three-way blends continued to show similar performance, albeit at a slightly higher error rate. As the number of three-way blends was huge, we showed the results obtained with common adulterants to groundnut oil; the 50th and the 90th percentile absolute errors were between 1.4 and 1.8% and 4–5.4%, respectively (Fig. 5). By contrast, traditional chemometric methods are not able to cope when complexities increase (Supplementary Fig. 3). In general, *n*-way blends can also be ascertained in a similar fashion but the practice of adulterating oils with too many components is impractical and lacks commercial incentive. Second, we also conducted a more realistic test to benchmark the model against real-life oil mixtures, measured with GC-FID. Across 46 groundnut oils, mixed with maize, sunflower and rice bran oil at varying degrees, the model performance had a median absolute error of 1.35% and 90th percentile absolute error of 2.7%. These results were very close to the simulated test error rates (Supplementary Table 5).

### Online-learning process

**Fig. 6:**
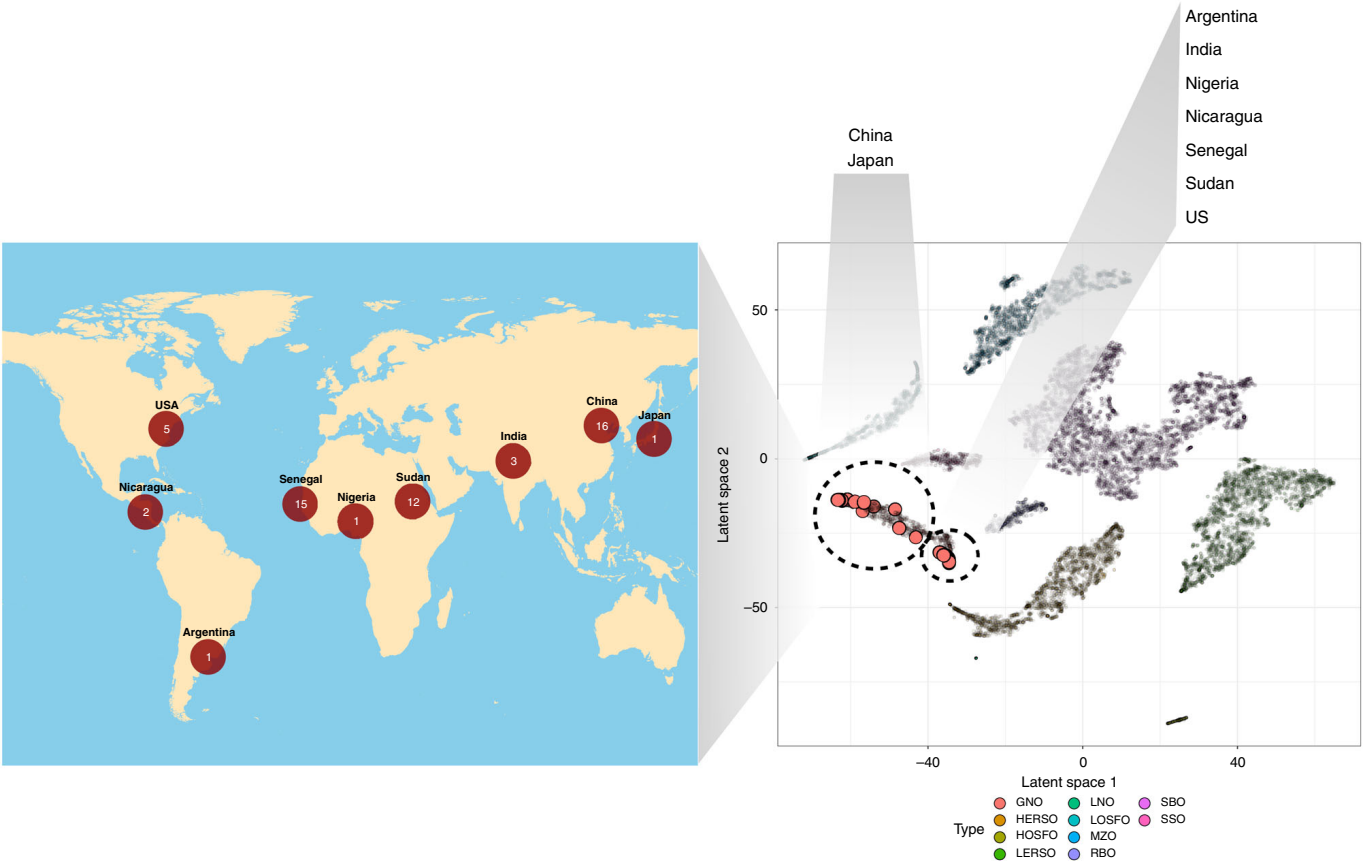
Groundnut oil for online-training. Fifty-six newly sourced divergent groundnut oils in the latent space used for online-training. Samples collected were geographically distributed as indicated on the map with the number of samples described in red circles.

Although we have demonstrated that the deep learning model performed well when benched mark against both simulated test sets as well as real-life blind mixtures, it is impossible to have a perfect training dataset due to biological variations of the same plant species being grown in, e.g., different geographical regions and subjected to varying environment conditions during growth. In order to evaluate the performance of the model built from oils that were sourced from production-line factories in China (old-world oils), we collected an additional 56 pure groundnut oils from various regions of China, India, Japan, North and South America, Africa and Middle East (new-world oils). Unsupervised analysis showed that in general these new oils clustered together with the groundnut oils from the initial dataset but displaying regional colocalizations (Fig. 6). Note that the Chinese and Japanese groundnut oils form a separate subcluster from the other groundnut oils. In addition, most of the new samples mapped to the boundaries of the larger groundnut oil cluster suggesting that it is possible for region specific fatty acid differences to introduce new information previously not detected.

The supervised deep learning model predicted a purity of between 91 and 99.5% of these newly surveyed groundnut oils. This deviation from the true purity could be due to the above-mentioned reasons supported by the evidence from unsupervised clustering around the edges of the large groundnut oil cluster. Consequently, we conducted two independent blind tests of real-life groundnut mixtures from two independent batches. We obtained a median absolute error of 3.4% and 4.75%, respectively, for the prediction of the major groundnut oil and 3.4% and 5.7%, respectively, for the prediction of minor adulterant oil. The 90th percentile absolute error was 7.54% and 8.24%, respectively for the major groundnut oil and 7.54% and 13.5%, respectively for the minor adulterant oil (Supplementary Table 6). An online-update of the deep learning model parameters was possible by supplying a smaller set of simulated oil mixtures made from the newly introduced pure oils^21,22^. This process improved the model prediction accuracy on real-life mixtures even though the actual fatty acid profiles of the mixed oil was not made known to the deep learning model. We found a median absolute error of 1.1 and 0.95% for predicting major groundnut oil (overall 64.8 and 61.9% reduction in errors, with 2.58 and 2.01% 90th percentile absolute error) and a median absolute error of 1.2 and 0.95% for predicting minor adulterant oil (overall 52.6 and 74.2% reduction in errors, with 3.04 and 2.1% 90th percentile absolute error, Supplementary Table 6). This update procedure assumes that the newly surveyed oils belong to one of the oil types in the preexisting database. In real life, completely new oil types can surface at a later stage. In order to account for this, multivariate clustering in the latent space can be used to identify new oil types (novel oils) before online-learning, for example cotton seed oil which was later added to the database shows as a separate cluster (Supplementary Fig. 4). Alternatively, the GMM parameters can also be used to compare the Mahalanobis distances between a new sample to the centroids representing each oil type in the database, for the detection of outlier oil types. The robustness of on-line learning applied to newly added oil types was also tested in our blind tests, where an actual groundnut oil mixed with cotton seed oil was quantified with small errors (Supplementary Table 6). These results demonstrate the utility of online-training to enhance performance and extend the general applicability to new and divergent oils.

## Discussion

Due to the increasing importance of plant oils in human diet, large databases have been established as a compilation of multiple published work^1,23^. Consequently, the availability of these databases has led to scientific endeavors to describe chemotaxonomic data on phylogenetics trees, characterizing the maximum yield of fatty acids across multiple taxa^23^. However, insightful study to these fatty acid profiles is still hampered by incomplete data across plant types (due to the compilation of different study designs) and the unavailability of a large quantity of samples. In addition, these characterization efforts also ignore multivariate changes in fatty acid composition across plant types, making inference of plant identities based on fatty acid profile difficult, and further encumbering the prediction of oil composition when a sample is presented as a mixture of multiple oils.

Utilizing a unified set of fatty acid profiles from a large number of oil samples collected by us, we conducted a comprehensive study to derive both inferential and predictive insights, enabled by the integration of machine learning technologies. Our analysis suggests that a new categorization of old-world plant oils is possible as revealed by machine learning patterns. These newly discovered subclusters of oil prompts further investigations to whether these observations are a consequence of natural biological evolution or human-assisted breeding efforts. Understanding of inter and intra variance between oil clusters also leads to the capabilities of simulating theoretical oil mixtures. The patterns generated from such mixtures was further exploited to construct a stronger supervised model capable of learning complex oil compositions.

Traditional supervised chemometric methods lack the feature of generalizability. Previous studies have shown that fatty acid profiles could be used to identify oil types via unsupervised clustering and supervised classification^9,10,12^ but these qualitative models were not directly generalizable to quantitative predictions. A recent work showed that a quantitative model for detecting two-way sesame oil mixtures showed promising results but exhibited higher errors when generalized to four-way mixtures^11^. Their results also could only be interpreted in the context of the nine chosen sesame oils used to create oil mixtures. Our supplementary experiments also show that generalizations of PLS led to higher errors when evaluated against our large dataset (Supplementary Tables 1–4). There are few existing methods that apply neural networks to fatty acid datasets, one such report was able to identify geographical origins of extra virgin olive oil^24^, but none that combines its discriminative power with large scale simulation for discovering oil composition. There are two added advantages of coupling the supervised deep learning machine to the simulation process: first, the simulation process is able to create independent mixtures for validating and optimizing the deep learning model through gradient descent. A third independent test set can also be easily generated to ensure that the model generalizes well to new unseen data. Second, when new oils are introduced, that are phenotypically different from those currently available, the simulation process can be used to guide updating of deep learning model parameters.

The utility of such predictions can be used in the area of food safety and security, specifically in the area of oil adulteration where oil prices across different plant oils are driven by market. Consumer driven edible oil consumption have resulted in a wide gap between the highly prized oil varieties and the less valued ones. In 2019, China consumed 3.09 million MT of groundnut oil priced at $14,500 CNY per metric ton^5^. Rarity also has an influence on price. Camellia tea seed oil which is almost exclusively produced in a region centered around the Yangtze river basin has a small production capacity of 0.26 million tons annually and is sold for $55,000 CNY per metric ton^25^. Olive oil, an oil with luxury status, is priced at $30,000 CNY per metric ton^26^. On the other hand, common oils like soybean, maize, and sunflower oil are sold for much cheaper at CNY6,850, CNY7,400, and CNY7,500 per metric ton, respectively^10,27^.

We foresee a larger scope of further studies in utilizing and extending the model to include many other new-world oils. Recent advances in physical technologies have also enabled rapid analytical systems that do not require sample preprocessing and therefore induce less chemical waste. For example, Fourier transform near infrared (FT-NIR) had been used with PLS1 in the detection of extra virgin olive oil adulteration^28^, Low-field nuclear magnetic resonance (LF-NMR) was used with discriminant analysis (DA) for the binary classification of adulterated peanut oil^29^, proton nuclear magnetic resonance was used with PLS1 for the detection of Camellia oil^30^ and excitation–emission matrix fluorescence spectroscopy had been used with N-way PLS for the detection of Camellia oil^31^. However, these studies also used traditional chemometric techniques and did not study the generalizability of their models across a large diverse set of samples. Our framework achieves this generalizability by incorporating a simulation module that scales up for complex mixtures with a deep learning model. Adaptation of such a workflow to these new technologies would be promising for future applications. The additional capabilities of online-training also provide a practical solution for incorporating new knowledge into the model. Nevertheless, leveraging on pretrained data, the utility of this model in quality control and assurance can provide a quick way to value purchased oils especially in regions where adulteration is prevalent. At a more basic level, the model can also be used to unify quality standards in the edible oil industry and also provide a more reliable way to label products and assisting food safety and security.

## Methods

### Sample collection

Oil samples of the ten oil types that make up the database for modeling were obtained from production plants in Yihai Kerry, China. These oil samples were derived from raw materials in the production plants to guarantee authenticity of purity and were collected over 5 years and across 30 factories in various provinces of China to account for biological variance. The groundnut oils used for real-life testing were crushed from raw materials sourced from USA, Nicaragua, Argentina, Senegal, Nigeria, Sudan, India, China, and Japan. These oil samples were processed in the laboratory with additional segregation in place so that samples can be traced down to a single origin.

### Fatty acid extraction and derivatization

Glycerol fatty acid ester were derivatized into FAMES according to AOCS Ce 2-66 by adding 8 ml of 2% sodium hydroxide in methanol solution, followed by 7 ml 15% boron-3-mofluororide in methanol solution at 80 degrees Celsius. Twenty milliliter of n-heptane and saturated sodium chloride solution were added to separate the mixture into organic and aqueous phase. Five milliliter of the upper organic layer was transferred into a 25 ml test tube, and 3–5 g of anhydrous sodium sulfate was added to absorb water, for subsequent gas chromatography analysis.

### Preparation of real-life oil mixtures

Groundnut oil, sunflower oil, high-oleic sunflower oil, maize oil, rice bran oil, and cottonseed oil were blended by different proportions determined using a Mettler Toledo electronic scale, with a precision of 0.1 mg.

### Gas chromatography flame ionization detector analysis

GC-FID was conducted with a column which fused silica capillary 100 m and 0.25 mm i.d. coated with SP-2560, 100% cyanopropylsilicone stationary phase to a thickness of 0.20 µm, with the following temperature programs. Initial temperature 100 °C, hold for 13 min; 100–180 °C with 10 °C/min, hold for 6 min; 180–200 °C with 1 °C/min, hold for 20 min; 200–230 °C with 4 °C/min, hold for 10.5 min. Carrier gas was nitrogen; inlet temperature: 270 °C; split ratio: 50:1; injection volume: 1.0 μl; FID detector temperature: 280 °C; FID detector hydrogen flow rate: 40 ml/min, air flow rate 400 ml/min, the makeup gas is nitrogen with a flow rate of 25 ml/min. Data was analyzed using Chemstation software to quantify each FAMEs with peak normalization method using ISO 12966-4-2015 standard.

### Data simulation and modeling

GC-FID fatty acid data were normalized to relative abundances and mapped to dimensionally reduced space using t-stochastic neighborhood embedding with Barnes–Hut approximation, perplexity hyperparameter was chosen based on the sample size of the dataset. Gaussian mixture model^26^ was fitted using expectation–maximization with the following model parameterizations: diagonal distribution, equal volume and shape and the number of clusters was optimized by Bayesian Information Criterion. Oil mixtures of two-way (^10^C_2_ combinations) and three-way (^10^C_3_ combinations) mixtures were a linear combination of simulated pure oil fatty acid profiles using the fitted GMM model, with weights corresponding to a random sample from Dirichlet distribution, the simulated samples were balanced by group. Deep learning models were trained with Keras^32^ using the TensorFlow^33^ backend, the optimizer, hyperparameters: lr and batch_size was tuned to reduce loss and training time without overfitting^27^. Normalized GC-FID fatty acid data was scaled using MinMax and used as inputs to the deep learning model, which comprises of a sequential stack of layers configured with a ReLu activation function and the kernel weights constrained to unit normal to prevent overfitting. PLS2 was performed using a NIPALS-based algorithm number of components was determined by tuning the R2Y scores and Q2Y scores and bootstrapping was performed to check for model overfitting by ensuring that Q2Y scores having a *p*-value of 0.05. PCA was performed based on SVD and data inputs were centered and scaled prior to analysis. All analysis was done using Python and R, using the following packages: Keras, TensorFlow, mclust, gtools, ropls, and FactomineR.

### Model evaluation

The GMM model was evaluated with the precision of each oil type *i* and sensitivity of each cluster *j*, these statistics are defined as:

1 $${\mathrm{Precision}}_{\mathrm{i}} = \frac{{\mathop {\sum}\nolimits_{c \in max_{\mathrm{i}}} {\left| {{\mathrm{Cluster}}_{\left. c \right\}}} \right|} }}{{|N_{\mathrm{i}}|}},$$

2 $${\mathrm{Sensitivity}}_{\mathrm{j}} = \frac{{|{\mathrm{Cluster}}_{\{ {\mathrm{maxj}}\} }|}}{{|{\mathrm{Mj}}|}},$$

where *N*_i_ refers to the number of samples of type *i*, max_i_ refers to the clusters which are maximally identified to be of type *i*, *M*_j_ refers to the number of samples in cluster *j* and max_j_ refers to the maximal oil type within cluster *j*.

The deep learning model was evaluated with independently simulated validation datasets. The absolute errors for two-way and three-way mixture predictions were vectorized and stratified by the ^10^C_2_ and ^10^C_3_ mixture types. The resulting 50th, 90th, 95th, and 99th percentile absolute errors were reported.

### Reporting summary

Further information on research design is available in the Nature Research Reporting Summary linked to this article.

## Supplementary information

Supplementary Information

Reporting Summary

## Data Availability

The data that support the findings of this study are available from Wilmar International and Yihai Kerry Arawana Holdings but restrictions apply to the availability of these data, which were used under license for the current study, and so are not publicly available. Data are however available from the authors upon reasonable request and with written permission from Wilmar International and Yihai Kerry Arawana Holdings, subject as the case may be to certain restrictions.

## Code availability

The code and models that are described in this study are available upon reasonable request and permission of Wilmar International. Requests for code access should be provided to Kevin Lim at the following email address: kevin.limjunliang@sg.wilmar-intl.com, who shall then communicate with the requester and consult with the company management on whether the requests meet the guidelines listed in the following. All codes are freely accessible for academic researchers provided that their projects are not supported by any commercial companies. Therefore, there might be requests from academic researchers who might be participating in projects supported by commercial entities whose requests may be denied.
