# Supplementary material for: Pattern recognition based on machine learning identifies oil adulteration and edible oil mixtures
Source: Nat Commun. 2020 Oct 23;11:5353. doi: 10.1038/s41467-020-19137-6 (PMC7584611; doi:10.1038/s41467-020-19137-6)
Supplement: Supplementary file 1 — Supplementary Information [file 41467_2020_19137_MOESM1_ESM.pdf]

Supplementary Information

Pattern recognition based on machine learning identifies oil adulteration and edible oil mixtures

Lim et. al.

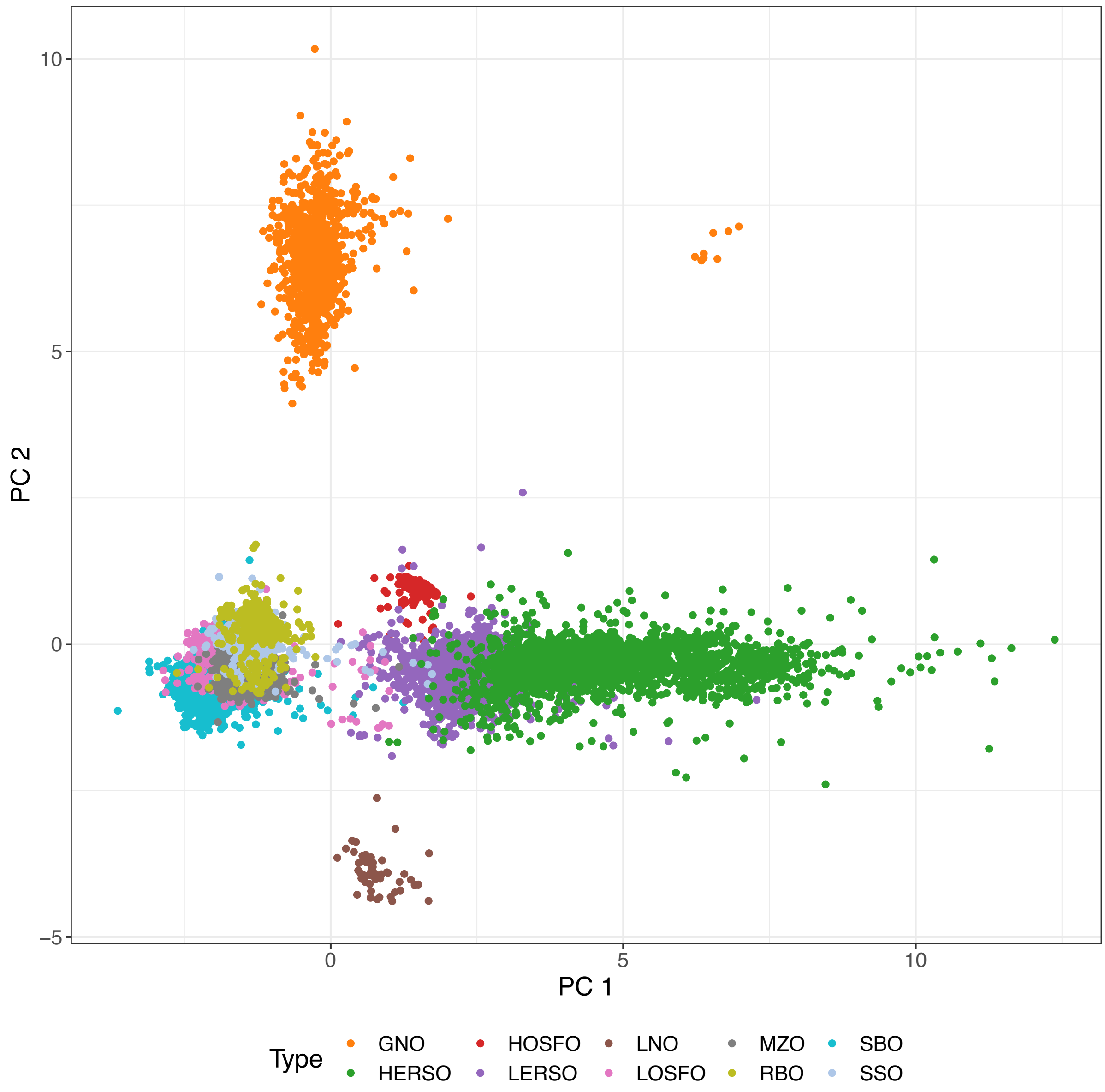

Supplementary Figure 1. Principal component analysis of the fatty acid profile of ten oil types.

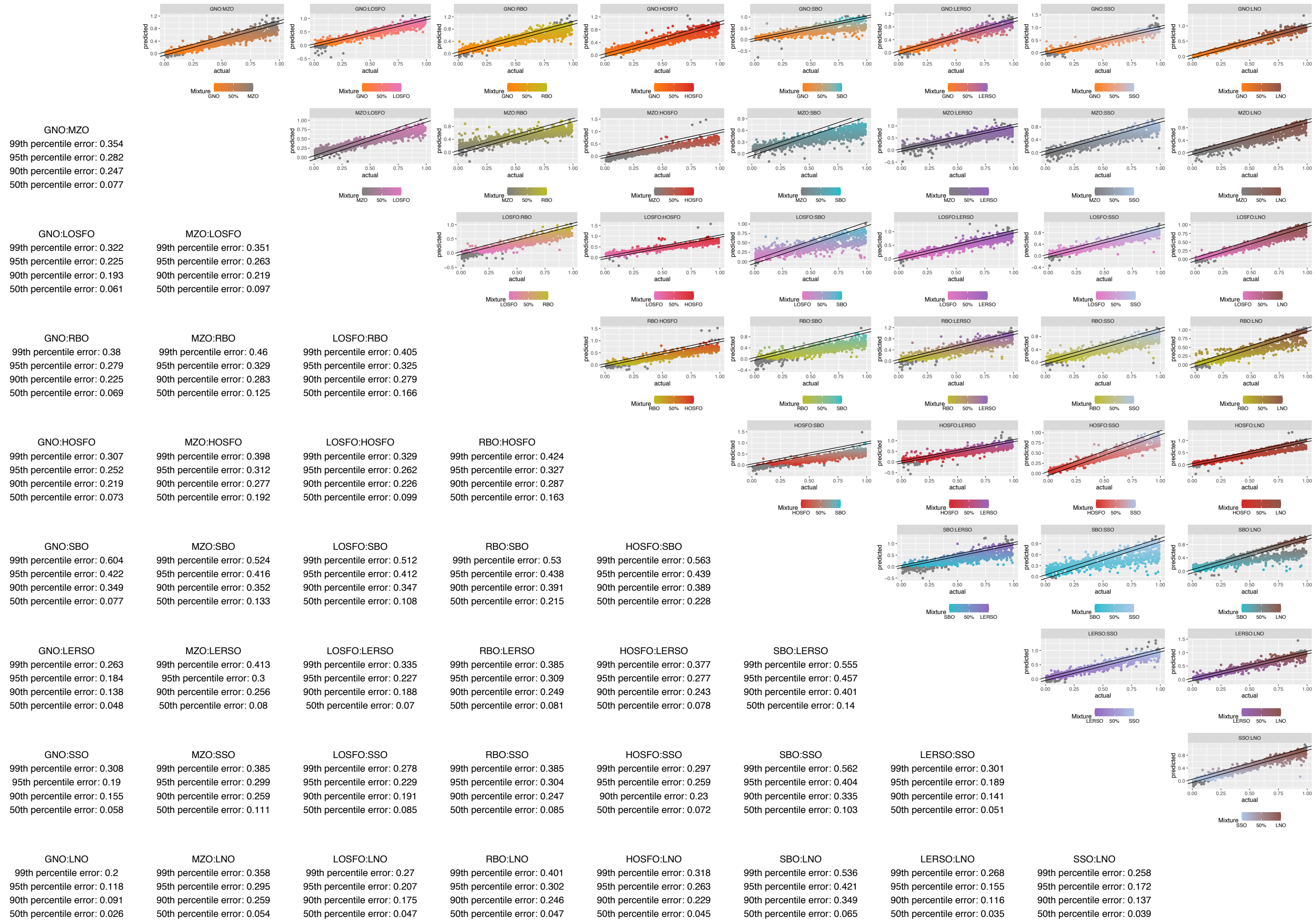

Supplementary Figure 2: Partial least squares prediction of 2-way mixtures showing all combinations of two-way mixtures of actual ratios against predicted ratios. The black lines indicate 5% margin of absolute error. Percentile absolute error is indicated in the lower triangle.

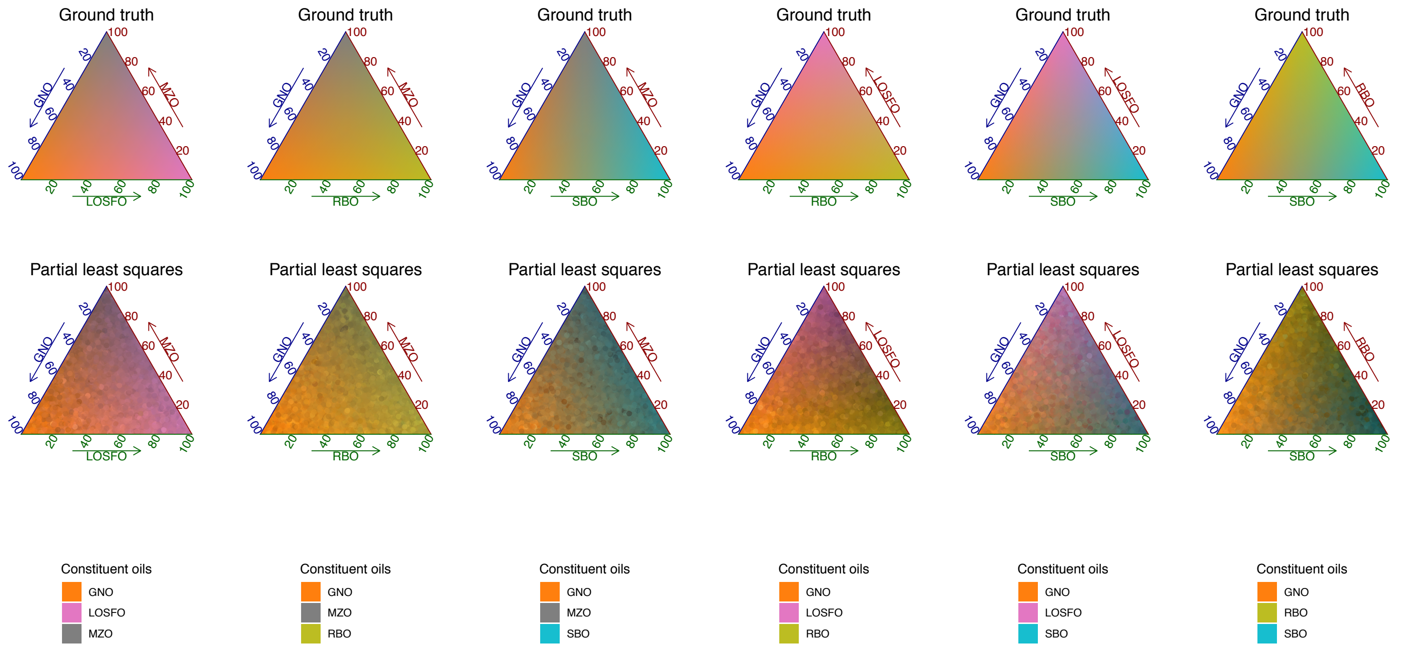

GNO:MZO:LOSFO	GNO:MZO:RBO	GNO:MZO:SBO	GNO:LOSFO:RBO	GNO:LOSFO:SBO	GNO:RBO:SBO
99th percentile error: 0.392	99th percentile error: 0.399	99th percentile error: 0.464	99th percentile error: 0.359	99th percentile error: 0.462	99th percentile error: 0.488
95th percentile error: 0.275	95th percentile error: 0.299	95th percentile error: 0.356	95th percentile error: 0.278	95th percentile error: 0.312	95th percentile error: 0.366
90th percentile error: 0.209	90th percentile error: 0.238	90th percentile error: 0.285	90th percentile error: 0.235	90th percentile error: 0.23	90th percentile error: 0.303
50th percentile error: 0.063	50th percentile error: 0.073	50th percentile error: 0.071	50th percentile error: 0.096	50th percentile error: 0.058	50th percentile error: 0.094

Supplementary Figure 3: Partial least squares prediction of 3-way mixtures, showing common adulterants to groundnut oil. (**TOP**) The ground truth represented by the Gibb's triangle, each point in the triangle correspond to exact ratios between three oils that sum to unity. The colors are the triangle represent the purity and the shift in color represents a mixture depending on the ratios between the other two oils. (**MIDDLE**) Results predicted by the 3-way PLS2 model are colored based on the relative ratios, colors that are closer to the ground truth have lower errors. (**BOTTOM**) Percentile absolute errors.

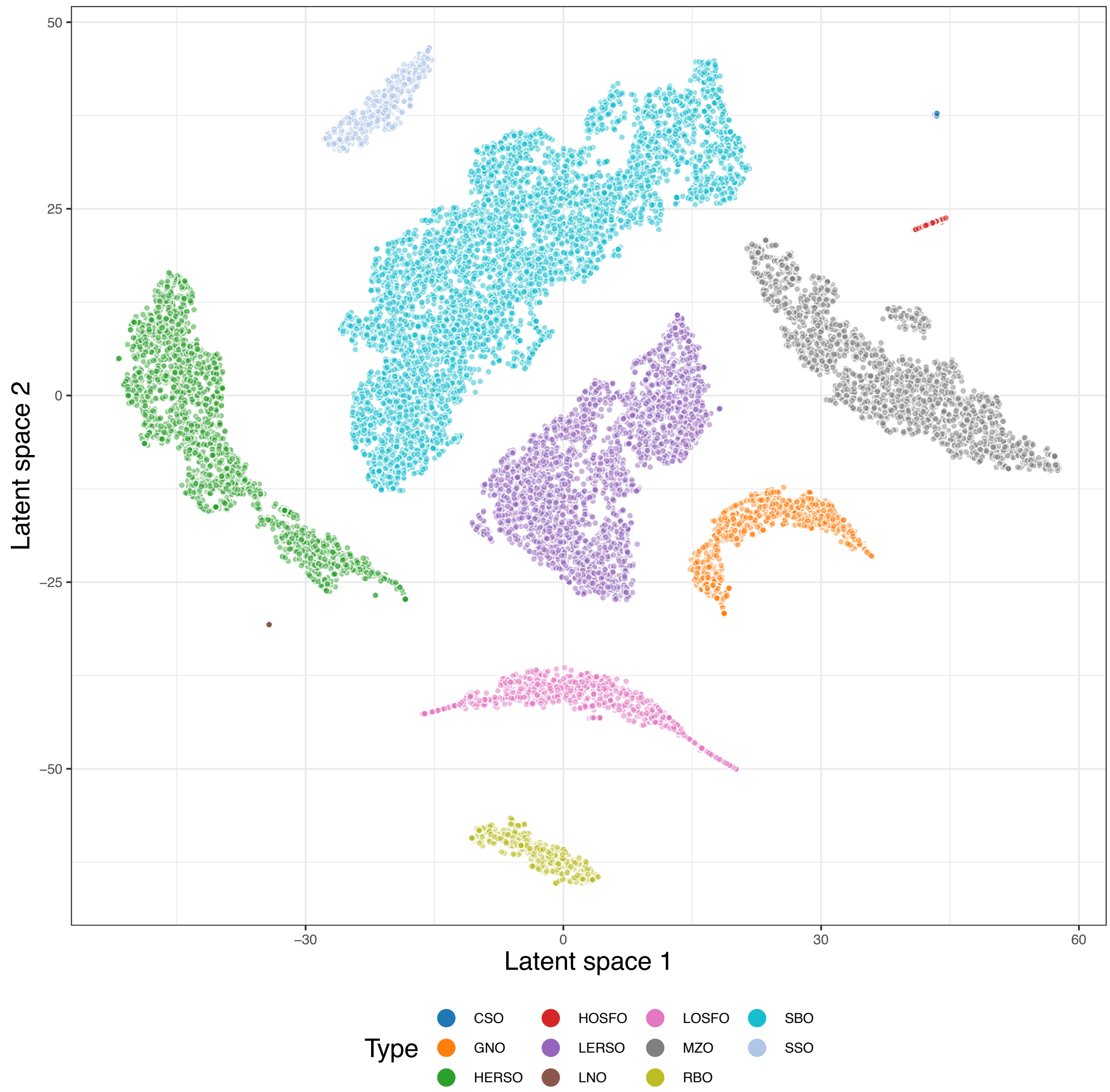

Supplementary Figure 4: Novel oil type (Cottonseed oil) form a separate cluster from old-world oils.

PLS1 with non-generalized test set									
50th percentile absolute error									
	GNO	MZO	LOSFO	RBO	HOSFO	SBO	LERSO	SSO	LNO
GNO	--	2.06%	1.98%	1.57%	1.28%	3.02%	1.98%	2.11%	1.38%
MZO	--	--	2.24%	2.61%	0.91%	3.48%	1.49%	2.14%	1.31%
LOSFO	--	--	--	1.10%	1.13%	2.70%	1.70%	2.79%	1.26%
RBO	--	--	--	--	0.69%	1.87%	1.16%	1.20%	1.34%
HOSFO	--	--	--	--	--	0.89%	1.27%	0.91%	0.75%
SBO	--	--	--	--	--	--	1.37%	3.03%	1.46%
LERSO	--	--	--	--	--	--	--	1.27%	1.70%
SSO	--	--	--	--	--	--	--	--	1.31%
LNO	--	--	--	--	--	--	--	--	--
90th percentile absolute error									
	GNO	MZO	LOSFO	RBO	HOSFO	SBO	LERSO	SSO	LNO
GNO	--	6.10%	5.52%	4.74%	4.61%	7.44%	5.43%	5.92%	3.99%
MZO	--	--	6.19%	7.01%	2.52%	10.07%	4.09%	6.38%	3.96%
LOSFO	--	--	--	3.17%	3.96%	6.94%	4.98%	7.70%	3.38%
RBO	--	--	--	--	1.91%	5.21%	4.01%	3.37%	3.66%
HOSFO	--	--	--	--	--	2.51%	3.69%	2.62%	1.93%
SBO	--	--	--	--	--	--	3.90%	8.20%	4.50%
LERSO	--	--	--	--	--	--	--	3.62%	3.92%
SSO	--	--	--	--	--	--	--	--	3.80%
LNO	--	--	--	--	--	--	--	--	--

Supplementary Table 1: Summary of 50th and 90th percentile errors for a PLS1 model with a non-generalized test set (Single-response model tested with only model specific mixtures), e.g. GNO:MZO model tested with GNO:MZO mixtures.

PLS1 with generalized test set									
50th percentile absolute error									
	GNO	MZO	LOSFO	RBO	HOSFO	SBO	LERSO	SSO	LNO
GNO	--	48.67 %	41.27 %	48.32 %	48.67 %	44.01 %	41.25 %	47.13 %	28.27 %
MZO	--	--	56.31 %	56.06 %	37.71 %	53.16 %	38.12 %	48.06 %	31.57 %
LOSFO	--	--	--	47.11 %	42.46 %	46.24 %	45.82 %	60.63 %	38.70 %
RBO	--	--	--	--	35.81 %	38.68 %	41.33 %	46.49 %	36.29 %
HOSFO	--	--	--	--	--	36.51 %	43.12 %	43.98 %	36.61 %
SBO	--	--	--	--	--	--	31.23 %	53.41 %	37.85 %
LERSO	--	--	--	--	--	--	--	40.79 %	37.24 %
SSO	--	--	--	--	--	--	--	--	33.31 %
LNO	--	--	--	--	--	--	--	--	--
90th percentile absolute error									
	GNO	MZO	LOSFO	RBO	HOSFO	SBO	LERSO	SSO	LNO
GNO	--	306.83 %	95.30 %	251.11 %	258.78 %	90.82 %	98.48 %	137.76 %	88.56 %
MZO	--	--	366.80 %	298.92 %	98.93 %	178.32 %	88.70 %	237.76 %	82.77 %
LOSFO	--	--	--	247.17 %	115.57 %	132.80 %	122.77 %	450.78 %	82.33 %
RBO	--	--	--	--	97.44 %	97.08 %	99.70 %	211.06 %	82.58 %
HOSFO	--	--	--	--	--	101.27 %	125.30 %	126.69 %	90.12 %
SBO	--	--	--	--	--	--	98.69 %	144.93 %	97.53 %
LERSO	--	--	--	--	--	--	--	86.46 %	84.14 %
SSO	--	--	--	--	--	--	--	--	95.29 %
LNO	--	--	--	--	--	--	--	--	--

Supplementary Table 2: Summary of 50th and 90th percentile errors for a PLS1 model with a generalized test set (Single-response model tested with non-model specific mixtures), e.g. GNO:MZO model tested with many other mixtures.

PLS2 with generalized test set									
50th percentile absolute error									
	GNO	MZO	LOSFO	RBO	HOSFO	SBO	LERSO	SSO	LNO
GNO	--	7.66%	6.12%	6.88%	7.34%	7.69%	4.82%	5.77%	2.65%
MZO	--	--	9.68%	12.54%	19.17%	13.29%	8.04%	11.09%	5.39%
LOSFO	--	--	--	16.56%	9.89%	10.79%	6.96%	8.51%	4.75%
RBO	--	--	--	--	16.28%	21.49%	8.12%	8.53%	4.72%
HOSFO	--	--	--	--	--	22.83%	7.77%	7.24%	4.55%
SBO	--	--	--	--	--	--	14.02%	10.26%	6.55%
LERSO	--	--	--	--	--	--	--	5.12%	3.46%
SSO	--	--	--	--	--	--	--	--	3.91%
LNO	--	--	--	--	--	--	--	--	--
90th percentile absolute error									
	GNO	MZO	LOSFO	RBO	HOSFO	SBO	LERSO	SSO	LNO
GNO	--	24.73%	19.29%	22.47%	21.95%	34.87%	13.78%	15.46%	9.07%
MZO	--	--	21.89%	28.27%	27.72%	35.17%	25.56%	25.94%	25.90%
LOSFO	--	--	--	27.85%	22.58%	34.72%	18.77%	19.13%	17.52%
RBO	--	--	--	--	28.67%	39.13%	24.91%	24.68%	24.63%
HOSFO	--	--	--	--	--	38.94%	24.35%	23.00%	22.91%
SBO	--	--	--	--	--	--	40.08%	33.51%	34.86%
LERSO	--	--	--	--	--	--	--	14.10%	11.60%
SSO	--	--	--	--	--	--	--	--	13.72%
LNO	--	--	--	--	--	--	--	--	--

Supplementary Table 3: Summary of 50th and 90th percentile errors for a PLS2 model with a generalized test set (Multi-response model tested with multi-mixtures).

Deep learning with generalized test set									
50th percentile absolute error									
	GNO	MZO	LOSFO	RBO	HOSFO	SBO	LERSO	SSO	LNO
GNO	--	1.23%	1.20%	1.03%	0.79%	1.47%	1.11%	1.12%	0.55%
MZO	--	--	1.45%	1.44%	0.65%	1.98%	1.01%	1.44%	0.51%
LOSFO	--	--	--	0.87%	0.79%	1.51%	1.00%	1.44%	0.52%
RBO	--	--	--	--	0.50%	1.35%	0.88%	0.95%	0.48%
HOSFO	--	--	--	--	--	0.70%	0.90%	0.60%	0.37%
SBO	--	--	--	--	--	--	1.13%	1.75%	0.71%
LERSO	--	--	--	--	--	--	--	0.90%	0.75%
SSO	--	--	--	--	--	--	--	--	0.53%
LNO	--	--	--	--	--	--	--	--	--
90th percentile absolute error									
	GNO	MZO	LOSFO	RBO	HOSFO	SBO	LERSO	SSO	LNO
GNO	--	3.38%	3.35%	2.89%	2.22%	4.04%	3.12%	3.12%	1.51%
MZO	--	--	4.05%	3.96%	1.79%	5.76%	3.00%	4.04%	1.46%
LOSFO	--	--	--	2.38%	2.64%	4.35%	2.85%	4.06%	1.51%
RBO	--	--	--	--	1.34%	3.63%	2.67%	2.59%	1.33%
HOSFO	--	--	--	--	--	1.94%	2.52%	1.64%	0.96%
SBO	--	--	--	--	--	--	3.56%	5.10%	1.93%
LERSO	--	--	--	--	--	--	--	2.68%	2.16%
SSO	--	--	--	--	--	--	--	--	1.52%
LNO	--	--	--	--	--	--	--	--	--

Supplementary Table 4: Summary of 50th and 90th percentile errors for a Deep learning with a generalized test set (Multi-response model tested with multi-mixtures).

S/N	Ground Truth (real oil mixtures)						Validation results							Absolute error wrt GNO (%)	Absolute error wrt adulterant oil (%)
	GNO	SFO	HOSFO	SSO	MZO	RBO	GNO	SFO	HOSFO	SSO	MZO	RBO	CSO		
1	95.00%	5.00%	0.00%	0.00%	0.00%	0.00%	95.20%	0.00%	4.80%	0.00%	0.00%	0.00%	0.00%	0.2	5
2	90.00%	10.00%	0.00%	0.00%	0.00%	0.00%	88.40%	11.60%	0.00%	0.00%	0.00%	0.00%	0.00%	1.6	1.6
3	85.00%	15.00%	0.00%	0.00%	0.00%	0.00%	83.40%	16.60%	0.00%	0.00%	0.00%	0.00%	0.00%	1.6	1.6
4	80.00%	20.00%	0.00%	0.00%	0.00%	0.00%	78.50%	21.50%	0.00%	0.00%	0.00%	0.00%	0.00%	1.5	1.5
5	70.00%	30.00%	0.00%	0.00%	0.00%	0.00%	70.10%	29.90%	0.00%	0.00%	0.00%	0.00%	0.00%	0.1	0.1
6	60.00%	40.00%	0.00%	0.00%	0.00%	0.00%	60.10%	39.90%	0.00%	0.00%	0.00%	0.00%	0.00%	0.1	-49.482
7	50.00%	50.00%	0.00%	0.00%	0.00%	0.00%	48.20%	51.80%	0.00%	0.00%	0.00%	0.00%	0.00%	1.8	1.8
8	95.00%	5.00%	0.00%	0.00%	0.00%	0.00%	97.60%	0.00%	0.00%	2.40%	0.00%	0.00%	0.00%	2.6	5
9	90.00%	10.00%	0.00%	0.00%	0.00%	0.00%	94.80%	5.20%	0.00%	0.00%	0.00%	0.00%	0.00%	4.8	4.8
10	85.00%	15.00%	0.00%	0.00%	0.00%	0.00%	87.80%	12.20%	0.00%	0.00%	0.00%	0.00%	0.00%	2.8	2.8
11	80.00%	20.00%	0.00%	0.00%	0.00%	0.00%	81.50%	18.50%	0.00%	0.00%	0.00%	0.00%	0.00%	1.5	1.5
12	70.00%	30.00%	0.00%	0.00%	0.00%	0.00%	69.90%	30.10%	0.00%	0.00%	0.00%	0.00%	0.00%	0.1	0.1
13	60.00%	40.00%	0.00%	0.00%	0.00%	0.00%	60.00%	40.00%	0.00%	0.00%	0.00%	0.00%	0.00%	0	0
14	50.00%	50.00%	0.00%	0.00%	0.00%	0.00%	48.80%	51.20%	0.00%	0.00%	0.00%	0.00%	0.00%	1.2	1.2
15	95.00%	0.00%	5.00%	0.00%	0.00%	0.00%	96.30%	0.00%	3.70%	0.00%	0.00%	0.00%	0.00%	1.3	1.3
16	90.00%	0.00%	10.00%	0.00%	0.00%	0.00%	91.00%	0.00%	9.00%	0.00%	0.00%	0.00%	0.00%	1	1
17	85.00%	0.00%	15.00%	0.00%	0.00%	0.00%	87.10%	0.00%	12.90%	0.00%	0.00%	0.00%	0.00%	2.1	2.1
18	80.00%	0.00%	20.00%	0.00%	0.00%	0.00%	82.00%	0.00%	18.00%	0.00%	0.00%	0.00%	0.00%	2	2
19	70.00%	0.00%	30.00%	0.00%	0.00%	0.00%	73.00%	0.00%	27.00%	0.00%	0.00%	0.00%	0.00%	3	3
20	60.00%	0.00%	40.00%	0.00%	0.00%	0.00%	62.50%	0.00%	37.50%	0.00%	0.00%	0.00%	0.00%	2.5	2.5
21	50.00%	0.00%	50.00%	0.00%	0.00%	0.00%	51.70%	0.00%	48.30%	0.00%	0.00%	0.00%	0.00%	1.7	1.7
22	95.00%	0.00%	5.00%	0.00%	0.00%	0.00%	95.10%	0.00%	4.90%	0.00%	0.00%	0.00%	0.00%	0.1	0.1
23	90.00%	0.00%	10.00%	0.00%	0.00%	0.00%	91.00%	0.00%	9.00%	0.00%	0.00%	0.00%	0.00%	1	1
24	85.00%	0.00%	15.00%	0.00%	0.00%	0.00%	85.70%	0.00%	14.30%	0.00%	0.00%	0.00%	0.00%	0.7	0.7
25	80.00%	0.00%	20.00%	0.00%	0.00%	0.00%	80.60%	0.00%	19.40%	0.00%	0.00%	0.00%	0.00%	0.6	0.6
26	70.00%	0.00%	30.00%	0.00%	0.00%	0.00%	70.70%	0.00%	29.30%	0.00%	0.00%	0.00%	0.00%	0.7	0.7
27	60.00%	0.00%	40.00%	0.00%	0.00%	0.00%	60.70%	0.00%	39.30%	0.00%	0.00%	0.00%	0.00%	0.7	0.7
28	50.00%	0.00%	50.00%	0.00%	0.00%	0.00%	51.10%	0.00%	48.90%	0.00%	0.00%	0.00%	0.00%	1.1	1.1
29	95.00%	0.00%	0.00%	0.00%	5.00%	0.00%	97.40%	0.00%	0.00%	0.00%	0.00%	2.60%	0.00%	2.4	5
30	90.00%	0.00%	0.00%	0.00%	10.00%	0.00%	93.90%	0.00%	0.00%	0.00%	6.10%	0.00%	0.00%	3.9	3.9
31	85.00%	0.00%	0.00%	0.00%	15.00%	0.00%	85.80%	0.00%	0.00%	0.00%	14.20%	0.00%	0.00%	0.8	0.8
32	80.00%	0.00%	0.00%	0.00%	20.00%	0.00%	80.70%	0.00%	0.00%	0.00%	19.30%	0.00%	0.00%	0.7	0.7
33	70.00%	0.00%	0.00%	0.00%	30.00%	0.00%	68.70%	0.00%	0.00%	0.00%	31.30%	0.00%	0.00%	1.3	1.3
34	60.00%	0.00%	0.00%	0.00%	40.00%	0.00%	59.20%	0.00%	0.00%	0.00%	40.80%	0.00%	0.00%	0.8	0.8
35	95.00%	0.00%	0.00%	0.00%	5.00%	0.00%	98.30%	0.00%	0.00%	0.00%	0.00%	1.70%	0.00%	3.3	5
36	90.00%	0.00%	0.00%	0.00%	10.00%	0.00%	92.20%	0.00%	0.00%	0.00%	7.80%	0.00%	0.00%	2.2	2.2
37	85.00%	0.00%	0.00%	0.00%	15.00%	0.00%	86.70%	0.00%	0.00%	0.00%	13.30%	0.00%	0.00%	1.7	1.7
38	80.00%	0.00%	0.00%	0.00%	20.00%	0.00%	81.40%	0.00%	0.00%	0.00%	18.60%	0.00%	0.00%	1.4	1.4
39	70.00%	0.00%	0.00%	0.00%	30.00%	0.00%	71.70%	0.00%	0.00%	0.00%	28.30%	0.00%	0.00%	1.7	1.7
40	60.00%	0.00%	0.00%	0.00%	40.00%	0.00%	62.60%	0.00%	0.00%	0.00%	37.40%	0.00%	0.00%	2.6	2.6
41	95.00%	0.00%	0.00%	0.00%	0.00%	5.00%	94.20%	0.00%	0.00%	0.00%	0.00%	5.80%	0.00%	0.8	0.8
42	90.00%	0.00%	0.00%	0.00%	0.00%	10.00%	89.10%	0.00%	0.00%	0.00%	0.00%	10.90%	0.00%	0.9	0.9
43	85.00%	0.00%	0.00%	0.00%	0.00%	15.00%	85.60%	0.00%	0.00%	0.00%	0.00%	14.40%	0.00%	0.6	0.6
44	95.00%	0.00%	0.00%	0.00%	0.00%	5.00%	96.40%	0.00%	0.00%	0.00%	0.00%	3.60%	0.00%	1.4	1.4
45	90.00%	0.00%	0.00%	0.00%	0.00%	10.00%	88.50%	0.00%	0.00%	0.00%	0.00%	11.50%	0.00%	1.5	1.5
46	85.00%	0.00%	0.00%	0.00%	0.00%	15.00%	85.40%	0.00%	0.00%	0.00%	0.00%	14.60%	0.00%	0.4	0.4

Supplementary Table 5: Evaluation results for old-world oils. 46 prospective real-world mixtures (5%-50%) from production-line was used for the blind test of the deep learning model.

Batch 1 (2020-03-17)

S/N	Ground Truth (real oil mixtures)						Validation results							Online Results (model refined with new pure oils, test mixtures hidden)											Error without online training	Error with online training	Error reduction	Error without online training	Error with online training	Error reduction
	GNO	MZO	SFO	CSO	RBO	HOSFO	GNO	SFO	HOSFO	SSO	MZO	RBO	CSO	GNO	HOSFO	CSO	SFO	MZO	RBO	CTO	SBO	RSO	SSO	LNO	Relative to GNO(%)	Relative to GNO(%)	Relative to GNO(%)	Relative to adulterant(%)	Relative to adulterant(%)	Relative to adulterant(%)
1	95.00	0.00	0.00	5.00	0.00	0.00	95.00	0.00	0.00	0.00	5.00	0.00	0.00	94.50	0.00	5.50	0.00	0.00	0.00	0.00	0.00	0.00	0.00	0.00	94.05	0.5	(99.47	5.00	0.5	(90.00
2	90.00	0.00	0.00	10.0	0.00	0.00	93.10	0.00	0.00	0.00	0.00	0.00	6.90	89.90	0.00	10.10	0.00	0.00	0.00	0.00	0.00	0.00	0.00	0.00	89.07	0.1	(99.89	9.93	0.1	(98.99
3	95.00	0.00	5.00	0.00	0.00	0.00	98.30	1.70	0.00	0.00	0.00	0.00	0.00	96.10	0.00	0.00	0.00	3.90	0.00	0.00	0.00	0.00	0.00	0.00	94.02	1.1	(98.83	4.98	5	0.34
4	90.00	0.00	10.00	0.00	0.00	0.00	94.50	5.50	0.00	0.00	0.00	0.00	0.00	90.90	0.00	0.00	9.10	0.00	0.00	0.00	0.00	0.00	0.00	0.00	89.06	0.9	(98.99	9.95	0.9	(90.95
5	85.00	0.00	15.00	0.00	0.00	0.00	90.00	10.00	0.00	0.00	0.00	0.00	0.00	86.70	0.00	0.00	13.3	0.00	0.00	0.00	0.00	0.00	0.00	0.00	84.10	1.7	(97.98	14.90	1.7	(88.59
6	80.00	0.00	20.00	0.00	0.00	0.00	84.30	15.70	0.00	0.00	0.00	0.00	0.00	81.80	0.00	0.00	18.2	0.00	0.00	0.00	0.00	0.00	0.00	0.00	79.16	1.8	(97.73	19.84	1.8	(90.93
7	70.00	0.00	30.00	0.00	0.00	0.00	73.60	26.40	0.00	0.00	0.00	0.00	0.00	70.20	0.00	0.00	29.8	0.00	0.00	0.00	0.00	0.00	0.00	0.00	69.26	0.2	(99.71	29.74	0.2	(99.33
8	60.00	0.00	40.00	0.00	0.00	0.00	62.50	37.50	0.00	0.00	0.00	0.00	0.00	58.90	0.00	0.00	41.1	0.00	0.00	0.00	0.00	0.00	0.00	0.00	59.38	1.1	(98.15	39.63	1.1	(97.22
9	50.00	0.00	50.00	0.00	0.00	0.00	52.20	47.80	0.00	0.00	0.00	0.00	0.00	48.20	0.00	0.00	51.8	0.00	0.00	0.00	0.00	0.00	0.00	0.00	49.48	1.8	(96.36	49.52	1.8	(96.37
10	95.00	0.00	0.00	0.00	0.00	5.00	99.10	0.90	0.00	0.00	0.00	0.00	0.00	97.10	2.90	0.00	0.00	0.00	0.00	0.00	0.00	0.00	0.00	0.00	94.01	2.1	(97.77	5.00	2.1	(58.00
11	90.00	0.00	0.00	0.00	0.00	10.0	98.30	1.70	0.00	0.00	0.00	0.00	0.00	90.80	9.20	0.00	0.00	0.00	0.00	0.00	0.00	0.00	0.00	0.00	89.02	0.8	(99.10	10.00	0.8	(92.00
12	85.00	0.00	0.00	0.00	0.00	15.0	95.10	4.90	0.00	0.00	0.00	0.00	0.00	85.70	14.3	0.00	0.00	0.00	0.00	0.00	0.00	0.00	0.00	0.00	84.05	0.7	(99.17	15.00	0.7	(95.33
13	80.00	0.00	0.00	0.00	0.00	20.0	91.10	8.90	0.00	0.00	0.00	0.00	0.00	81.00	19.0	0.00	0.00	0.00	0.00	0.00	0.00	0.00	0.00	0.00	79.09	1	(98.74	20.00	1	(95.00
14	70.00	0.00	0.00	0.00	0.00	30.0	77.50	0.00	22.5	0.00	0.00	0.00	0.00	71.10	28.9	0.00	0.00	0.00	0.00	0.00	0.00	0.00	0.00	0.00	69.23	1.1	(98.41	29.78	1.1	(96.31
15	60.00	0.00	0.00	0.00	0.00	40.0	66.70	0.00	33.3	0.00	0.00	0.00	0.00	62.10	37.9	0.00	0.00	0.00	0.00	0.00	0.00	0.00	0.00	0.00	59.33	2.1	(96.46	39.67	2.1	(94.71
16	50.00	0.00	0.00	0.00	0.00	50.0	55.60	0.00	44.4	0.00	0.00	0.00	0.00	52.50	47.5	0.00	0.00	0.00	0.00	0.00	0.00	0.00	0.00	0.00	49.44	2.5	(94.94	49.56	2.5	(94.96
17	95.00	5.00	0.00	0.00	0.00	0.00	98.00	0.00	0.00	0.00	2.00	0.00	0.00	95.90	0.00	0.00	0.00	4.10	0.00	0.00	0.00	0.00	0.00	0.00	94.02	0.9	(99.04	4.98	0.9	(81.93
18	90.00	10.0	0.00	0.00	0.00	0.00	95.80	0.00	0.00	0.00	4.20	0.00	0.00	89.30	0.00	0.00	0.00	10.7	0.00	0.00	0.00	0.00	0.00	0.00	89.04	0.7	(99.21	9.96	0.7	(92.97
19	85.00	15.0	0.00	0.00	0.00	0.00	91.30	0.00	0.00	0.00	8.70	0.00	0.00	84.60	0.00	0.00	0.00	15.4	0.00	0.00	0.00	0.00	0.00	0.00	84.09	0.4	(99.52	14.91	0.4	(97.32
20	80.00	20.0	0.00	0.00	0.00	0.00	86.90	0.00	0.00	0.00	13.1	0.00	0.00	79.60	0.00	0.00	0.00	20.4	0.00	0.00	0.00	0.00	0.00	0.00	79.13	0.4	(99.49	19.87	0.4	(97.99
21	70.00	30.0	0.00	0.00	0.00	0.00	78.10	0.00	0.00	0.00	21.9	0.00	0.00	69.60	0.00	0.00	0.00	30.4	0.00	0.00	0.00	0.00	0.00	0.00	69.22	0.4	(99.42	29.78	0.4	(98.66
22	60.00	40.0	0.00	0.00	0.00	0.00	67.50	0.00	0.00	0.00	32.5	0.00	0.00	60.10	0.00	0.00	0.00	39.9	0.00	0.00	0.00	0.00	0.00	0.00	59.33	0.1	(99.83	39.68	0.1	(99.75
23	90.00	0.00	0.00	0.00	10.0	0.00	89.40	0.00	0.00	0.00	10.6	0.00	0.00	91.00	0.00	0.00	0.00	0.00	9.00	0.00	0.00	0.00	0.00	0.00	89.11	1	(98.88	10.00	1	(90.00
24	80.00	0.00	0.00	0.00	20.0	0.00	80.60	0.00	0.00	0.00	19.4	0.00	0.00	78.70	0.00	0.00	0.00	0.00	21.3	0.00	0.00	0.00	0.00	0.00	79.19	1.3	(98.36	20.00	1.3	(93.50

Batch 2 (2020-04-12)

S/N	Ground truth (real oil mixtures)						Validation results							Online Results (model refined with new pure oils, test mixtures hidden)											Error without online training	Error with online training	Error reduction	Error without online training	Error with online training	Error reduction
	GNO	MZO	SFO	CSO	RBO	HOSFO	GNO	SFO	HOSFO	SSO	MZO	RBO	RSO	GNO	MZO	SFO	CSO	RBO	CTO	HOSFO	SBO	RSO	SSO	LNO	Relative to GNO(%)	Relative to GNO(%)	Relative to GNO(%)	Relative to adulterant(%)	Relative to adulterant(%)	Relative to adulterant(%)
25	95.00	0.00	5.00	0.00	0.00	0.00	87.70	0.00	0.00	12.3	0.00	0.00	0.00	94.60	5.40	0.00	0.00	0.00	0.00	0.00	0.00	0.00	0.00	0.00	94.12	0.4	(99.58	5.00	5	0.00
26	90.00	0.00	10.00	0.00	0.00	0.00	81.40	18.60	0.00	0.00	0.00	0.00	0.00	89.60	0.00	10.40	0.00	0.00	0.00	0.00	0.00	0.00	0.00	0.00	89.19	0.4	(99.55	9.81	0.4	(95.92
27	85.00	0.00	15.00	0.00	0.00	0.00	82.80	17.20	0.00	0.00	0.00	0.00	0.00	85.60	0.00	14.40	0.00	0.00	0.00	0.00	0.00	0.00	0.00	0.00	84.17	0.6	(99.29	14.83	0.6	(95.95
28	80.00	0.00	20.00	0.00	0.00	0.00	78.30	21.70	0.00	0.00	0.00	0.00	0.00	81.40	0.00	18.60	0.00	0.00	0.00	0.00	0.00	0.00	0.00	0.00	79.22	1.4	(98.23	19.78	1.4	(92.92
29	70.00	0.00	30.00	0.00	0.00	0.00	67.50	32.50	0.00	0.00	0.00	0.00	0.00	70.50	0.00	29.50	0.00	0.00	0.00	0.00	0.00	0.00	0.00	0.00	69.33	0.5	(99.28	29.68	0.5	(98.32
30	95.00	5.00	0.00	0.00	0.00	0.00	94.70	0.00	0.00	2.00	3.30	0.00	0.00	94.90	5.10	0.00	0.00	0.00	0.00	0.00	0.00	0.00	0.00	0.00	94.05	0.1	(99.89	4.97	0.1	(97.99
31	90.00	10.0	0.00	0.00	0.00	0.00	87.70	0.00	0.00	0.00	12.3	0.00	0.00	88.40	11.6	0.00	0.00	0.00	0.00	0.00	0.00	0.00	0.00	0.00	89.12	1.6	(98.20	9.88	1.6	(83.80
32	85.00	15.0	0.00	0.00	0.00	0.00	81.80	0.00	0.00	0.00	18.2	0.00	0.00	83.50	16.5	0.00	0.00	0.00	0.00	0.00	0.00	0.00	0.00	0.00	84.18	1.5	(98.22	14.82	1.5	(89.88
33	80.00	20.0	0.00	0.00	0.00	0.00	77.00	0.00	0.00	0.00	23.0	0.00	0.00	78.30	21.7	0.00	0.00	0.00	0.00	0.00	0.00	0.00	0.00	0.00	79.23	1.7	(97.85	19.77	1.7	(91.40
34	70.00	30.0	0.00	0.00	0.00	0.00	65.60	0.00	0.00	0.00	34.4	0.00	0.00	68.90	31.1	0.00	0.00	0.00	0.00	0.00	0.00	0.00	0.00	0.00	69.34	1.1	(98.41	29.66	1.1	(96.29
35	95.00	0.00	0.00	0.00	5.00	0.00	93.30	0.00	0.00	2.50	4.20	0.00	0.00	94.20	0.00	0.00	0.00	5.80	0.00	0.00	0.00	0.00	0.00	0.00	94.07	0.8	(99.15	5.00	0.8	(84.00
36	90.00	0.00	0.00	0.00	10.0	0.00	87.00	0.00	0.00	0.00	0.00	13.0	0.00	90.80	0.00	0.00	0.00	9.20	0.00	0.00	0.00	0.00	0.00	0.00	89.13	0.8	(99.10	9.87	0.8	(91.89
37	85.00	0.00	0.00	0.00	15.0	0.00	82.00	0.00	0.00	0.00	0.00	18.0	0.00	86.20	0.00	0.00	0.00	13.8	0.00	0.00	0.00	0.00	0.00	0.00	84.18	1.2	(98.57	14.82	1.2	(91.90

38	95.00	5.00	0.00	0.00	0.00	0.00	90.90	9.10	0.00	0.00	0.00	0.00	0.00	93.80	6.20	0.00	0.00	0.00	0.00	0.00	0.00	0.00	0.00	0.00	94.09	1.2	(98.72	5.00	1.2	(76.00
39	90.00	10.0	0.00	0.00	0.00	0.00	81.30	18.70	0.00	0.00	0.00	0.00	0.00	89.70	10.3	0.00	0.00	0.00	0.00	0.00	0.00	0.00	0.00	0.00	89.19	0.3	(99.66	10.00	0.3	(97.00
40	85.00	15.0	0.00	0.00	0.00	0.00	77.70	0.00	0.00	0.00	22.3	0.00	0.00	84.30	15.7	0.00	0.00	0.00	0.00	0.00	0.00	0.00	0.00	0.00	84.22	0.7	(99.17	14.78	0.7	(95.26
41	80.00	20.0	0.00	0.00	0.00	0.00	74.40	0.00	0.00	0.00	25.6	0.00	0.00	79.60	20.4	0.00	0.00	0.00	0.00	0.00	0.00	0.00	0.00	0.00	79.26	0.4	(99.50	19.74	0.4	(97.97
42	70.00	30.0	0.00	0.00	0.00	0.00	66.60	0.00	0.00	0.00	33.4	0.00	0.00	69.90	30.1	0.00	0.00	0.00	0.00	0.00	0.00	0.00	0.00	0.00	69.33	0.1	(99.86	29.67	0.1	(99.66
43	95.00	0.00	5.00	0.00	0.00	0.00	87.40	12.60	0.00	0.00	0.00	0.00	0.00	91.70	0.00	8.30	0.00	0.00	0.00	0.00	0.00	0.00	0.00	0.00	0.00	94.13	3.3	(96.49	4.87	3.3	(32.29
4	90.0	0.0	10.0	0.0	0.0	0.0	84.1	15.9	0.0	0.0	0.0	0.0	0.0	86.9	0.0	13.1	0.0	0.0	0.0	0.0	0.0	0.0	0.0	0.0	0.0	89.1	3.1	(96.5	9.84	3.1	(68.5
4	85.0	0.0	15.0	0.0	0.0	0.0	80.3	15.9	0.0	0.0	0.0	0.0	0.0	82.2	0.0	17.8	0.0	0.0	0.0	0.0	0.0	0.0	0.0	0.0	0.0	84.2	2.8	(96.6	14.84	2.8	(81.1
4	80.0	0.0	20.0	0.0	0.0	0.0	75.9	24.1	0.0	0.0	0.0	0.0	0.0	81.2	0.0	18.8	0.0	0.0	0.0	0.0	0.0	0.0	0.0	0.0	0.0	79.2	1.2	(98.4	19.76	1.2	(93.9
4	70.0	0.0	30.0	0.0	0.0	0.0	68.6	31.4	0.0	0.0	0.0	0.0	0.0	68.8	0.0	31.2	0.0	0.0	0.0	0.0	0.0	0.0	0.0	0.0	0.0	69.3	1.2	(98.2	29.69	1.2	(95.9

Supplementary Table 6: Evaluation results and online learning improvements for new-world oils. 47 prospective real-world mixtures (5%-50%) from new-world oils were first blind tested from the deep learning model trained with retrospective data. The online training procedure learns from the pure new-world oils using the schematic in Figure 3, but the real-world mixture data is withheld and preserved for a second blind test.
